# 3D mapping of intra-articular calcaneal fractures

**DOI:** 10.1038/s41598-023-34711-w

**Published:** 2023-05-31

**Authors:** Guang Shi, Zhao Lin, Wei Liu, Xun Liao, Xingming Xu, Xue Luo, Hongrui Zhan, Xiyu Cai

**Affiliations:** 1 Department of Orthopedics, The Fifth Affiliated Hospital of Sun Yat-Sen University, Zhuhai, 519000 Guangdong China; 2 Department of Rehabilitation, The Fifth Affiliated Hospital of Sun Yat-Sen University, Zhuhai, 519000 Guangdong China

**Keywords:** Computer modelling, Computer science, Anatomy

## Abstract

To determine the pattern of intra-articular calcaneal fractures (ICFs) by a three-dimensional (3D) mapping and determine whether there were consistent fracture patterns and comminution zones*.* In this study, 67 patients with ICFS by CT scan were included. The calcaneal fractures fragments in CT were multiplanar reconstructed and virtual reduced. 3D heat mapping was subsequently created by graphically superimposing all fracture lines onto a standard calcaneal template. The cohort included 26 (38.8%) left calcaneal fractures, 27 (40.30%) right calcaneal fractures, and 14 (20.9%) cases with bilateral fractures. Comminuted fractures accounted for 92.5%. Sagittal 3D mapping shows that the fracture line is mainly concentrated at the critical angle of Gissane and extending rear to the posterior of the tuberosity of the lateral wall and the anterior of the medial process of the calcaneus tuberosity but with more significant variation in the medial wall. The average angle of fracture lines concerning the long calcaneal axis (LCA) was 29.1° and 19.2° in the lateral and medial walls. Axial 3D mapping shows that fracture lines were primarily concentrated in the anterior area to the posterior joint facet and extending along the rear joint facet and calcaneus sulcus to the posteriorly of the tuberosity. The mean angle of fracture lines concerning the LAC was 11° in the axial wall. Our data provided elucidated that ICFs have consistent characteristic fracture patterns and comminution zones. This study provides visual guidelines for understanding fracture morphology, which may assist with fracture classification, preoperative planning, development of fixation concepts.

## Introduction

Calcaneal fracture accounts for about 2% of all fractures, which is the most common fracture of tarsal bone; more than 60% of cases are related to axial load^1^. Extra-articular fractures can exist in isolation or as a portion of intra-articular injuries, and intra-articular fractures are more common, accounting for about 75% of adult calcaneal fractures^2,3^. Due to the complexity of the fracture patterns, there is a high incidence of complications, including wound infection, plantar fasciitis, and post-traumatic arthritis. The management of ICFs remains controversial.

The complexity and variability of ICFS make understanding and managing these fractures challenging. Understanding these fracture patterns is the basis for proper management. Initial evaluation of suspicious calcaneal fractures is usually performed with an X-ray. However, its visualization of the calcaneal anatomy is limited, and the X-ray is commonly insufficient to determine a surgical approach^4^. Based on the complex anatomy of ICFs, CT is frequently recommended and permitted surgeons to realign the fracture fragments, fix them with plates or screws, and restore the subtalar joint facet^5,6^. Nevertheless**, **the widely utilized and popularized Sanders classification is based on CT but only moderate intra-observer reliability^7^. Moreover, these classifications cannot involve the 3D morphological structure of calcaneal fractures.

Displaced ICFs are complex injuries. The essential factors for the successful management of ICFs include visualizing the articular surface injury and understanding fracture patterns. These requirements can only be achieved through in-depth knowledge of the fracture pattern. There remains much controversy regarding the location and direction of the calcaneal fracture line^8,9^. Recently, with the rapid development of mapping technology and digital medical processing software, a new method, 3D mapping was proposed to define the distribution of fracture lines and comminuted zones. Armitage et al. were early to describe fracture lines characterize with 3D CT^10^. Yao et al.^11^ used 3D mapping to demonstrate the typical pattern of fractures and verify the rationalization of classification. This fracture mapping improves our understanding of fracture patterns and morphology^12,13^. We consider that 3D mapping would reveal more detailed information than previous X-ray and CT.

To the best of our knowledge, fracture mapping has previously been performed on complex ICFs^14^. However, Ni et al. did not describe the correlation between the common comminution zones and fracture lines in ICFs. We aim to determine the location and frequency of fracture lines and comminution zones of ICFs by 3D mapping techniques. These 3D maps intended to inform surgeons of the likely locations of fracture lines and comminution zones of ICFs involvement. We hypothesize that insight into patterns of ICFs may promote our understanding of this complex fracture, and there are consistent fracture patterns and comminution zones.

## Methods

### Subjects

This study was approved by the ethics committee of the fifth affiliated Hospital of Sun Yat-sen University. Between January 2019 and December 2020, a total of 182 adult patients with closed calcaneal fractures were enrolled. The International Classification of Diseases (ICD-9-CM) codes to identify calcaneal fractures. Inclusion criteria: (1) intra-articular calcaneal fracture; (2) Patients over 18 years of age; (3) preoperative CT scans with adequate quality. Exclusion criteria: (1) pathological fractures; (2) previous surgery; (3) old fractures; (4) open fractures. In total, 67 of 182 patients with ICFs were screened into this study. All fractures were identified using the Sanders classifications. Case records were retrieved to identify the concomitant fracture.

### Radiological analysis

**Figure 1:**
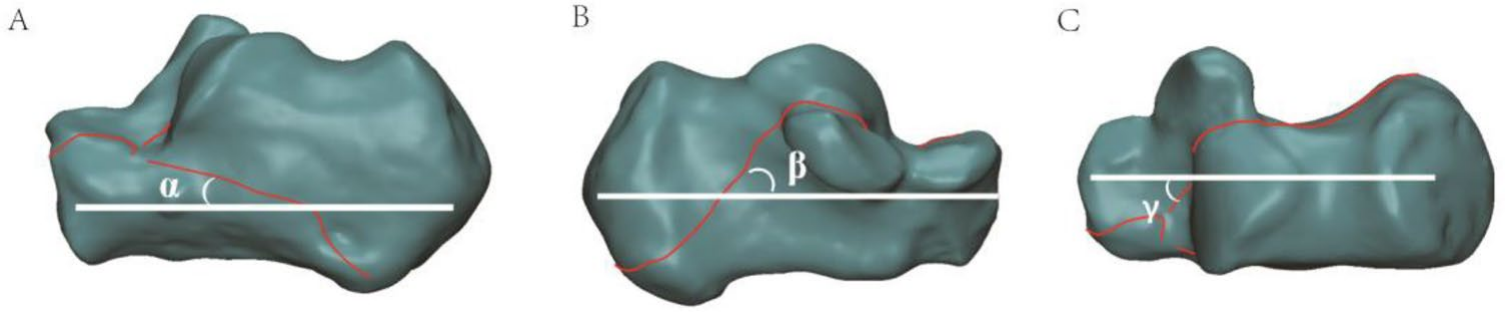
3D measurements of calcaneal fracture lines. (**A**–**C**) Angle α, β, and γ were defined as the acute angle formed by the fracture line (red line) with the long calcaneal axis (LCA) (white line). Image from Mimics software and 3-Matic 13.0 software.

All patients have performed CT scans (Siemens, Berlin, Germany), and the data was saved as Digital Imaging and Communication in Medicine (DICOM) files. The raw CT data was transferred to Mimics21.0 software (Materialise). Then reconstruct the 3D model to analyze the fracture lines from the axial and sagittal planes-the long calcaneal axis (LCA) as a reference line. The angle α and angle β were determined as the acute angle formed by the fracture line and LCA (Fig. 1A,B). The value was defined as positive when the fracture lines were from the anterior superior to the inferior posterior aspect of the calcaneal; otherwise, negative value was obtained. Angle γ was determined as the acute angle formed by the fracture lines and the LCA (Fig. 1C). The value was defined as positive when the fracture line was from anterolateral to posteromedial in the calcaneal; otherwise, negative value was obtained.

### Fracture mapping

**Figure 2:**
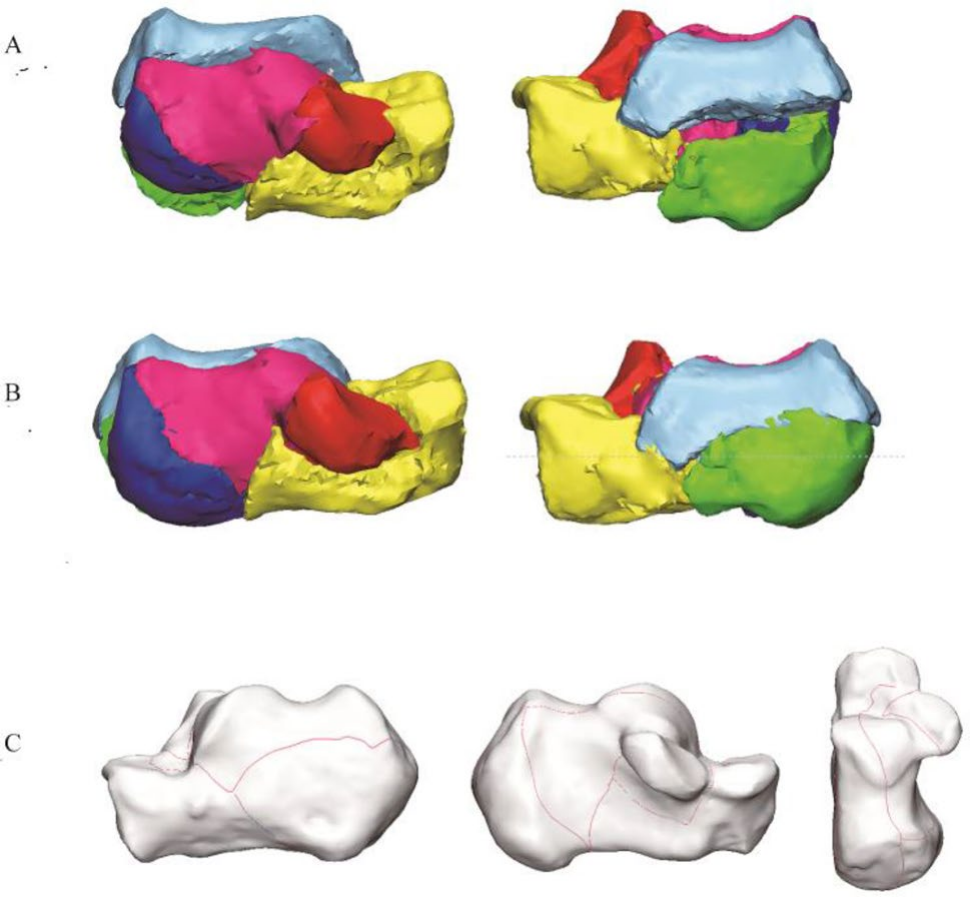
The method used for the mapping of the calcaneal fracture. (**A**) Each fragment was reconstructed. (**B**) Fracture fragments were virtually reduced. (**C**) Fracture line was marked with a red line on the standard template. Image from Mimics software and 3-Matic 13.0 software.

**Figure 3:**
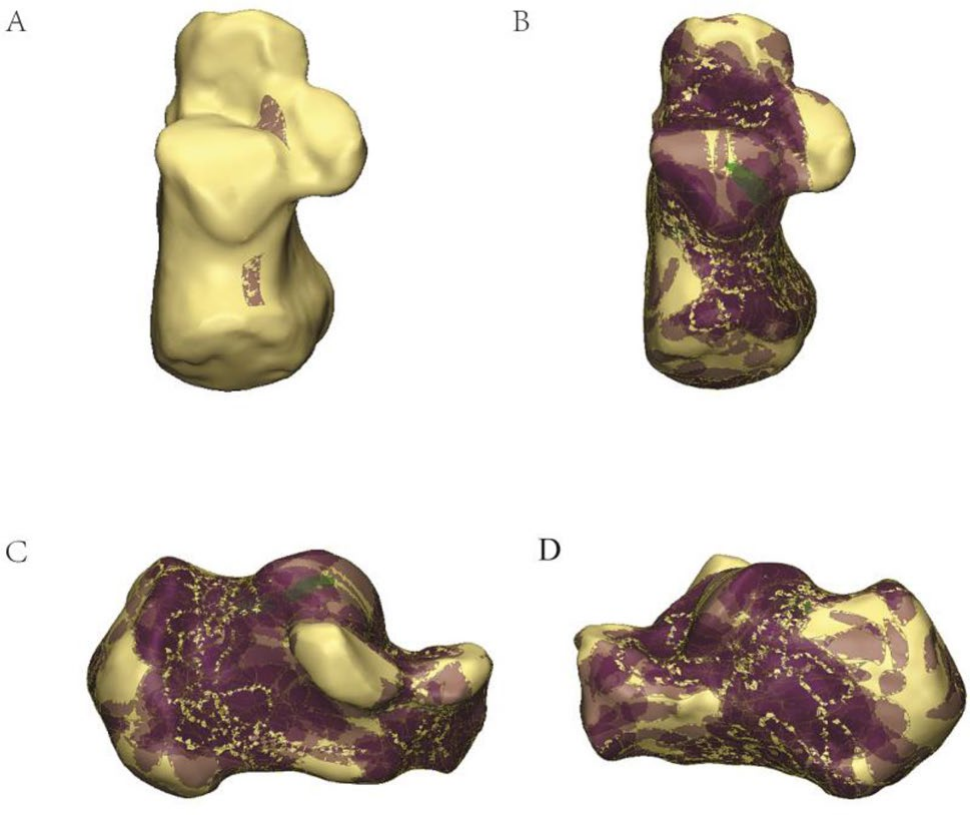
The method used for the mapping of the calcaneal fractures. (**A**) Marking comminution zones on the calcaneal template. (**B**–**D**) All comminution zones were superimposed on the template. Purple color represents a higher frequency of comminution zones density. Image from Mimics software and 3-Matic 13.0 software.

**Figure 4:**
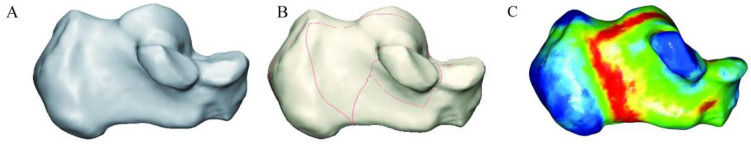
The method used for the mapping of calcaneal fractures. (**A**) Intact calcaneal template. (**B**) Fracture lines were delineated on the template. (**C**) 3D heat mapping of calcaneal fracture lines. Image from e-3D software (Central South University Changsha, China).

The calcaneal fracture fragments were 3D reconstructed in Mimics software and virtual reduced. (Fig. 2). Subsequently, data were imported into the 3-Matic 13.0 software. The reconstructed fragments were standardized, rotated, flipped, mirror to best match a 3D model of the standard template calcaneus. Smooth curves were depicted precisely on the calcaneus template surface to delineate each case's fracture line and comminution zones distribution in 3-Matic (Fig. 3). If necessary, closure curves may be applied. All fracture lines and comminution zones were transferred into e-3D software (Central South University Changsha, China) to generate 3D heat maps (Fig. 4). On the 3D heat maps, different colours graphically represent the different frequencies of fractures and comminution^11^.

### Data analysis

Analysis of 3D mapping was descriptive^10^. Qualitative data were presented as the number (percentage), and quantitative data were expressed as the mean (SD) using SPSS 21.0. Summarize patient characteristics and fracture measurements as mean and standard deviation or proportion.

### Declarations

We confirm that all methods were performed in accordance with the relevant guidelines and regulations.

### Ethics approval and consent to participate

This study was approved by the local ethics committee (The Fifth affiliated Hospital of Sun Yat-sen University). Data were analyzed retrospectively; informed consent was obtained from all participants.

## Results

**Figure 5:**
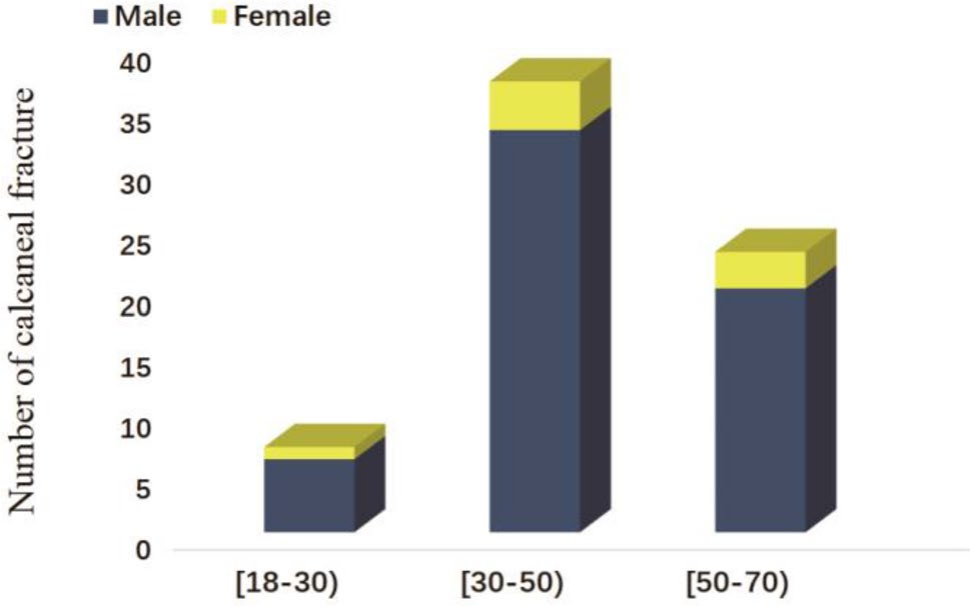
The distribution of fractures by patient age.

Patient characteristics, radiological classification, and 3D model measurements of the ICFs were summarized in Tables 1 and 2. We retrospectively reviewed records of 182 Patients in this study, 67 Patients of ICFs with available CT scans were identified. Among the 67 patients with 81 ICFs were included. Including 26 (38.8%) left calcaneal fractures, 27 (40.30%) right calcaneal fractures, and 14 (20.90%) cases with bilateral fractures. Among the 67 patients, the mean age was 44.50 years (SD 11.80). Fifty-nine were males (88.06%), and eight were female (11.94%), and fractures are most common to occur among patients aged 30–70 years and are more common in males (88.06%) (Fig. 5). 60 (89.60%) were caused by falling incidents, and 7 (10.40%) were caused by a traffic incident. The Sanders classification of the cohort was as follows: Sanders I, 14 (17.28%); Sanders II, 25 (30.86%); and Sanders III, 24 (2.63%); Sanders IV, 18 (22.23%).

**Table 1:** Patient demographics.

Demographic data (n = 67)	
Mean age, years (SD)	
Male	43.98 (10.87)
Female	48.00 (17.33)
Total	44.46(11.80)
Sex, n (%)	
Male	59 (88.05)
Female	8 (11.95)
Fractures, n (%)	
Left only	26 (38.8%)
Right only	27 (40.30%)
Bilateral	26 (38.8%)
Total	67 (100.0)
Concomitant fractures (no. [%])	
Fracture	35(52.24)
No	32(47.76)

**Table 2:** Fracture characteristics and classification.

Fracture characteristics and classification	
Sanders classification, n (%)	
I	14 (17.28%)
II	25 (30.86%)
III	26 (38.8%)
IV	18 (22.23%)
Fracture patterns, n (%)	
Comminution	62 (92.5)
Simple	5 (7.5)
Fracture mechanism, n (%)	
Falling incidents	60 (89.6)
Traffic incidents	7 (10.4)
Angle (°)	
Lateral α	29.1 ± 36.9
Medial β	19.2 ± 42.4
Coronal γ	11.0 ± 35.4

### 3D mapping

**Figure 6:**
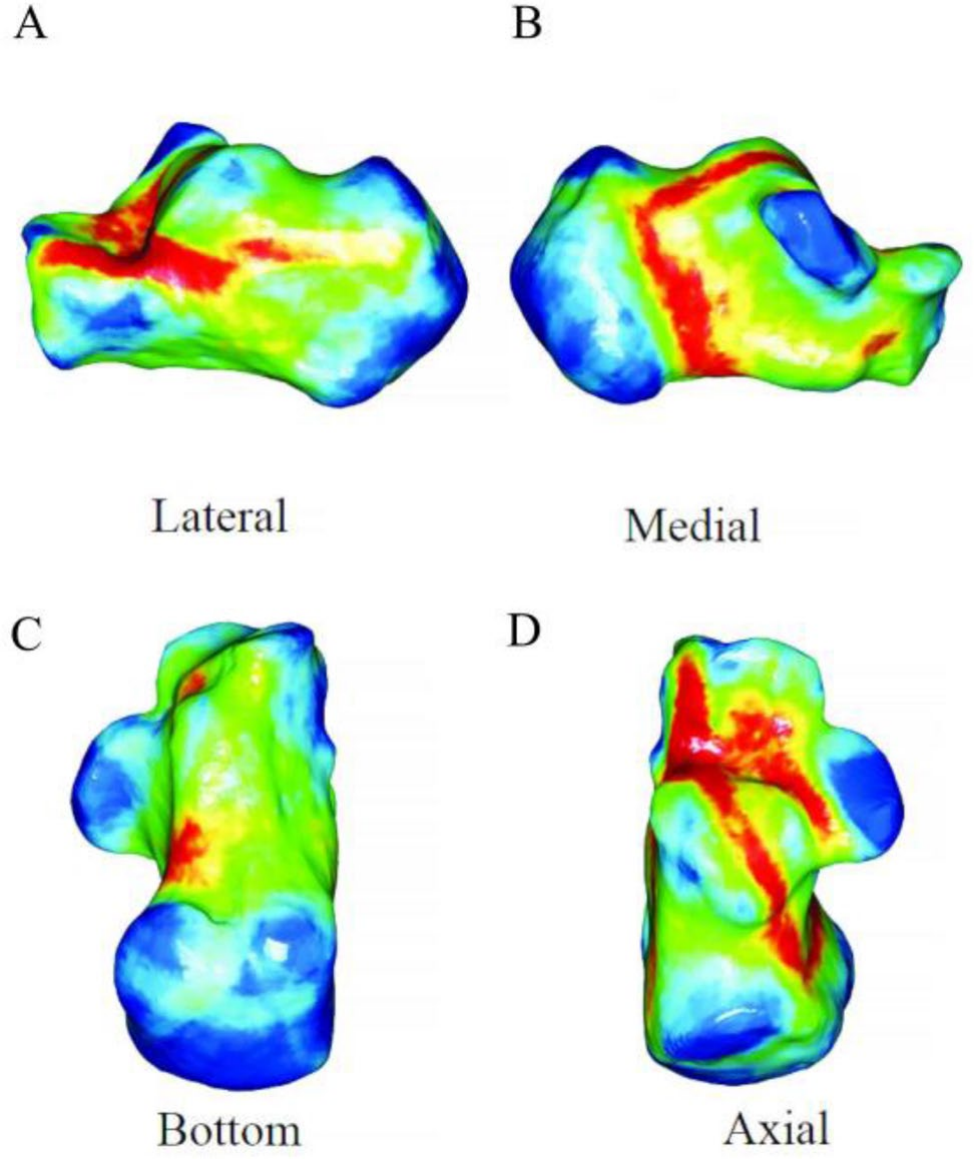
(**A**–**D**) Representative views of all fracture lines were superimposed on the template. (**F**–**I**) 3D heat mapping superimposed with all fracture lines, including the lateral, medial, bottom, axial views. The red color represents a higher frequency of fracture line density. Image from e-3D software (Central South University Changsha, China).

The 67 patients' fracture lines were superimposed on a standard calcaneal template. On 3D heat mapping (Fig. 6A–D), the hot zones were primarily concentrated at the critical angle of Gissane and extending rear to the posterior of the tuberosity, the anterior region of the medial process of the calcaneal tuberosity and extend partly to the bottom of the calcaneus and partly in the direction of the calcaneocuboid joint (CCJ), the anterior area to the posterior articular surface and extending along the posterior joint facet and calcaneus sulcus to the rear of the tuberosity. The frequency of fracture lines in the above areas were most intensive. Moreover, the anterior of the posterior facet joint was predisposed to a substantial amount of fracture lines and comminution zones. The cold zones of fracture lines were scattered in the sustentaculum tali, the calcaneus's anterior process, and the posterior calcaneus tuberosity. The ICFs have consistent fracture patterns and comminuted areas.

### Axial maps

**Figure 7:**
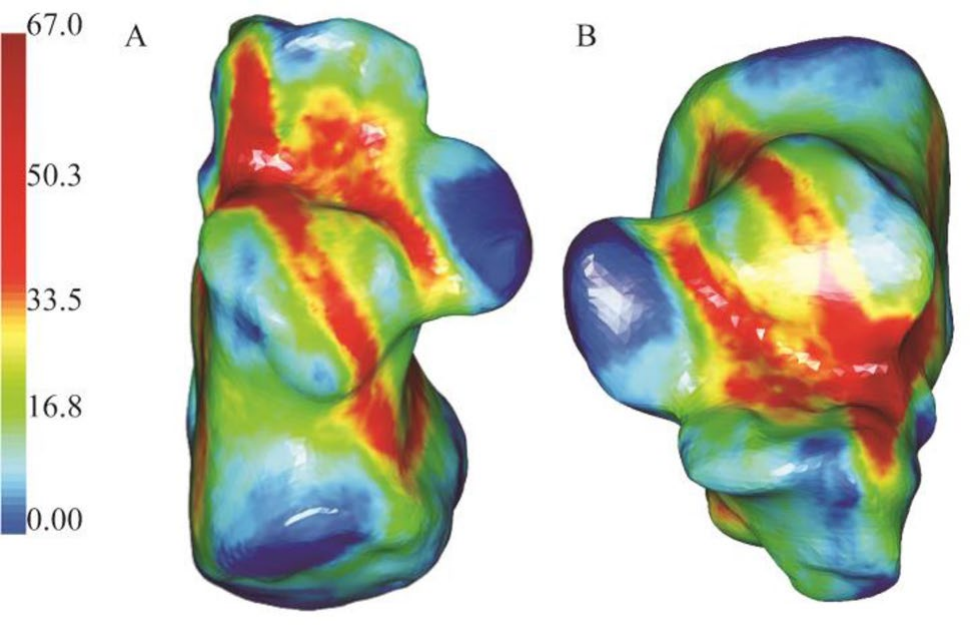
(**A**,**B**) Representative axial 3D mapping of fracture lines of the intra-articular calcaneal. fractures. Image from e-3D software (Central South University Changsha, China).

On axial fracture 3D mapping, the hot zones of the fracture lines were primarily concentrated in the anterior area of the posterior articular surface and extended along the posterior joint facet and calcaneus sulcus to the posteriorly of the tuberosity (Fig. 7). The mean γ angle measurement was 11.0° (range − 82.86° to 84.46°). The comminution zones' hot zones also were mainly located in the anterior area of the posterior joint facet and extended medially to the calcaneal tuberosity.

### Sagittal maps

**Figure 8:**
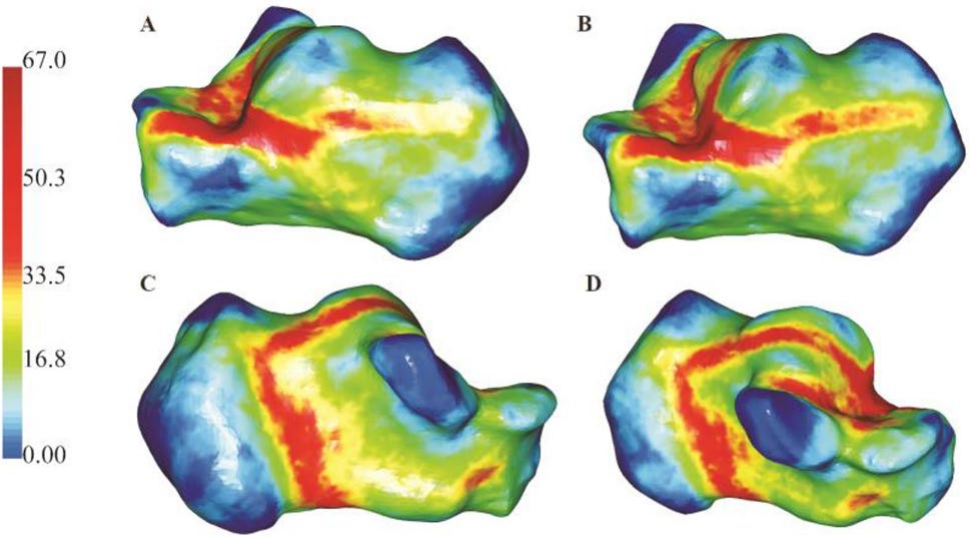
(**A**,**B**) Representative sagittal 3D mapping of fracture lines of the intra-articular calcaneal fractures. (**A**,**B**) Lateral. (**C**,**D**) Medial. Image from e-3D software (Central South University Changsha, China).

On the surface of the lateral wall of the calcaneus, the hot zones of fracture lines were located at the critical angle of Gissane and extended to the rear of the tuberosity. It was a typical characteristic of an inverted “Y” pattern (Fig. 8). The mean α angle measurement was 29.1° (range − 71.45° to 73.99°). The comminution zones were commonly located at the critical angle of the Gissane and the middle of the lateral wall of the calcaneal, posteriorly below the peroneal muscle trochlea. The hot zones of the medial wall were primarily concentrated in the anterior region of the medial process of the calcaneal tuberosity and extend partly to the bottom of the calcaneus and partly in the direction of the CJJ. The mean β angle measurement was 19.2° (range − 82.53° to 87.95°). The comminution zones also were frequently located in the middle part of the medial wall and the inferior one-third aspect of the medial of the CCJ.

## Discussion

The 3D mapping technique was described by Cole et al. and Mellema et al.^15,16^. In the current study, we perform 3D mapping technology representing the distribution and frequency of fracture lines and comminuted areas of ICFs. The overall 3D mapping of ICFs showed that the fracture lines mainly concentrated at the critical angle of Gissane, extended along the laterally to posteriorly and affected the lateral wall, the anterior area of the posterior joint facet, developed along the posterior joint facet and calcaneus sulcus, and extended posteriorly, medially to affect the posterior aspect and cortical walls. The anterior of the rear facet joint was predisposed to plenty of fracture lines and comminution zones. Moreover, the research shows that ICFs have consistent fracture patterns and comminuted zones. These results reveal the calcaneal's internal structure characteristics, provided more detailed information than traditional X-ray or CT, indicating that the distribution of fracture lines of ICFs related to the internal anatomical structure.

Several studies have described the distribution and orientation of the calcaneus fracture lines^17,18^. The primary fracture lines cut the calcaneal into two parts, medial and lateral^8,9^. Carr et al.^19^ reported that the fracture line typically separated the posterior facet and extended anteriorly to involve the anterior cuboid facet and could extend medially to affect the middle facet. Essex-Lopresti suggests that the anterolateral process of the talus generated the primary fracture lines to separate the middle facet. Warrick et al.^20^ reported that the main fracture lines extend posterior medial of the sustentacular fragment, with differences in extension distance. Recently, Tsubone et al. predicted fracture lines of the calcaneal by a 3D finite element model. They showed that the fracture line always starts from the lateral of the posterior joint fragment and extends in the anteromedial and anterolateral directions. Ni et al.^14^ showed that the fracture lines of ICFs were mainly concentrated in the calcaneus sulcus, extended medially, rear, anteriorly to affect the posterior facet surface and cortical walls. In addition, One study reported that the medial wall of the sustentacular and tuberosity fragments at the fracture site often had comminuted zones^21^. In the current study, the fracture lines and comminution zones were regularly distributed along the calcaneal surface. The majority of the fracture lines were located in the critical angle of Gissane and the area anterior to the posterior joint facet and extended along the posterior joint facet and calcaneus sulcus to the posteriorly of the tuberosity and was a typical characteristic of inverted “Y” pattern in the lateral wall. These findings have led to a better understanding of the pattern of calcaneal fractures, allowing for a better selection of surgical incisions and fixation methods.

The distribution of the fracture lines and comminution zones correlates well with the calcaneal's internal submicroscopic structure and biomechanics. The calcaneus is the most prominent tarsal bone, which provides elastic but forceful support for the body's weight, with a thin cortical shell surrounding cancellous bone^22,23^. Athavale et al.^22^ proved that the weaker zones of calcaneal and emphasize the primary influence of the internal architecture in predicting the fracture lines. Chen et al.^24^ perform a finite element model to evaluate the biomechanical of locking plates, showed that the fragments at the posterior articular surface and the posterior tuberosity sustained more stress. Xu et al.^25^ reported in another study the loading force was transmitted primarily by the anteroinferior portion, remarkably close to the bottom of the sinus tarsi, with the significant contact regions on the lateral, anterior and posterior sides. Wong et al.^26^ evaluated the influence of foot impingement on the risk and location of calcaneal fracture by finite element model, and stresses were primarily in the angle of Gissane and posterior articular surface. One study reported that knowledge of the weak areas can improve the technique of internal fixation^22^. By 3D heat maps, the study to be consistent with previous studies. These more vulnerable zone’s location indicates that the area should be avoided when screw fixation is applied. Furthermore, the fracture lines and comminution areas of ICFs revealed in our study may improve the fixation concepts.

The calcaneus fractures rarely involve the sustentaculum tali. Several studies have proven that sustentaculum tali is a ‘‘constant fragment’’^21,27,28^. However, Heger et al. evaluated 25 patients with calcaneal fractures and reported the sustentacular fractures in eighteen^29^. Della Rocca et al. evaluated more than 300 cases of calcaneal fractures treated with surgery and found 19 cases of sustentacular fractures^30^. The present study found that 21 (25.9%) fracture lines passed into the sustentaculum tali. Our findings are consistent with the work of Heger et al. and Della Rocca et al. in that patient with ICFs involved the sustentacular fragment that challenges the notion of anatomic constancy as a ‘‘constant fragment’’. We also found that no comminution in the sustentaculum tali. Therefore, it provides an effective position for screw fixations. Although our study is a 3D reconstruction of superimposed all fracture lines in a standard template. Any comparison of the outcome should be cautions because of subtle methodologic differences. Moreover, the isolated lateral approach is based on the “constant” nature of the sustentacular fragments^31^. The idea that the fragment is “constant” invites an alternative surgical approach that should be improved. Berberian et al. said it seems reasonable to consider a medial approach or combined medial and lateral approaches when the sustentaculum tali is seen to be fractured on preoperative CT scans^31^. So, we think a CT scanning should be routinely performed when a suspected fracture is found in the sustentacular fractures.

Calcaneal fractures caused by axial load is the most common^2^. However, the mean angle of fracture lines concerning the LCA was 29.1 (range − 71.45° to 73.99°) and 19.2 (range − 71.45° to 73.99°) in the lateral wall and medial wall. The vertical fracture line is rare. We also demonstrated that fracture lines distribution in the anterior process of the calcaneus was relatively rare, and the CCJ was involved with the fracture lines consistent with Ni et al.^14^. In addition, we found that the comminution zones tend to involve the inferior one-third aspect of the medial of the CCJ. Several studies have confirmed the probability of CCJ involvement in calcaneal fractures ranges from 33 to 76%^32–34^. Previous studies have shown that poor reduction of CCJ can lead to impingement symptoms or lateral peritalar subluxation^35^. Many studies relied on X-rays only and cannot routinely perform CT scans^35^. In the 3D heat maps, the comminution zones were located on the inferior one-third aspect of the medial of the CCJ surface. These might not be apparent in traditional radiology, so CT scanning should be routinely performed for patients with calcaneal fractures.

3D mapping can help develop a more comprehensive classification system. Earlier classification systems for calcaneal fractures were based on traditional X-rays; the Essex-Lopresti system is the best known^2^. This study provided a good description of the mechanism of injury and the orientation of the fracture line and helped identify extra-articular injuries and intra-articular injuries. However, to our knowledge, visualization of the calcaneal anatomy and specific comminution zones at conventional X-ray is limited. The involvement of the subtalar articular surface and medial wall cannot reflect by Essex Lopresti classification. Moreover, the interobserver reliability among radiologists was poor for the Essex-Lopresti classification (kappa = 0.26)^36^. In this study, we found that the fracture lines directions are continuous variables. Designating the location of fracture lines as dichotomous variables in the form of the classification systems will never result in an entirely consistent result*.* We believe that the subjective classification of fracture patterns into two types described by Essex Lopresti et al. may not obtain satisfactory interobserver agreement. Therefore, the calcaneus classification based on X-ray findings is obsolete. In 1993, based on coronal and axis, CT images of the Sanders classification are the most used system for classifying ICFs^37^, subdividing into four types, depending on the number of fractures and the position of fracture lines at the posterior calcaneal facet^5^. Despite its being widely used, the value of this classification is always disputed due to its limited reliability and validity^38^. This system does not consider fracture displacement in the sagittal or axial plane relative to the widest undersurface of the posterior talar articular surface and the pathological changes of calcaneal fractures. Therefore, a new classification that can reflect morphological changes and damage to the subtalar articular surface should be seriously considered. In the current study, the areas with the highest concentration of fracture lines and comminuted zones were described by 3D mapping. The orientation of the fracture lines and the location of the comminuted zones, which can be accurately reflected. The fracture mapping can provide clearer, more accurate information as well as enhancing our understanding^39^. Compared with previous anatomical and radiological reports, the present 3D mapping provides more detailed information and may prove helpful in facilitating improved comminuted zones and morphology understanding of classification concepts to manage complex ICFs injuries better.

Improved understanding of ICFs morphology and fracture lines by 3D heat maps may facilitate preoperative planning and development of fixation concepts. Several biomechanical studies comparisons the advantage of indifferent fixed ways^24,40,41^. To adapt to the anatomical and biomechanical characteristics of the subtalar and CCJ, plate fixation may be a good option^42^. Plate fixation using a sinus tarsi approach, which is currently popular, directly reduction the articular surface through the incision^43^. This approach was the most popular minimally approach for treatment of calcaneal fractures. However, in the sagittal plane, the fracture line always points to the critical angle of Gissane and extends posteriorly the calcaneal tuberosity of the lateral wall. The most concentration of fracture lines was slightly below the tarsal sinus approach. This fracture line’s location indicates that when fixation from the lateral wall, the screws should be positioned to avoid this area as much as possible and potentially suggests that a lower preoperative incision approach would be better.

Although there are important discoveries by these studies, there are also limitations to be considered. First, patients with insufficient CT data were excluded. The exclusion of these patients resulted in a statistical error in fracture incidence. Second, the number of included patients was relatively small, and the more accurate the results might be if the larger the number of cases. Third, the methods and results were descriptive, and one may argue that the interpretation of 3D mapping is subjective. Fourth, because of the limitations of 3D mapping technology, some reconstructed models cannot well match the 3D calcaneal model. The fracture lines and comminution zones superposition on the calcaneus model might be subtly different. Finally, due to the virtual reduction procedure, the existing 3D heat maps technique can only show the distribution of the fracture line and comminution zones on the calcaneal surface rather than the displacement and compression of the fragments. We also cannot more rational computations or analyses on the stress or stress intensity factors of those fracture patterns. However, our study also had important strengths. To our knowledge, we are the first to apply the 3D mapping technique^11^ to describe the correlation between the common comminution zones and fracture lines in ICFs.

## Conclusions

The data provided elucidated that ICFs have consistent characteristic fracture patterns and comminution zones. This study provides visual guidelines for understanding fracture morphology, which may assist with fracture classification, preoperative planning, development of fixation concepts, and internal structure analysis.

## Data Availability

The datasets generated and analyzed during the current study can be available from the corresponding author on reasonable request.

## Abbreviations

ICFs: Intra-articular calcaneal fractures
3D: Three-dimensional
CT: Computed tomographic
LCA: Long calcaneal axis
CCJ: Calcaneocuboid joint
